# Ballroom dance and positive aging among middle-aged and older adults: a chain mediation model of social connection and loneliness

**DOI:** 10.3389/fpsyg.2026.1855200

**Published:** 2026-05-29

**Authors:** Dongquan Gao, Xiaojie Sun, Nur Shakila Mazalan, Denise Koh

**Affiliations:** 1 National University of Malaysia, Bangi, Malaysia; 2 Hebei Chemical and Pharmaceutical College, Shijiazhuang, China

**Keywords:** ballroom dance, chain mediation, loneliness, positive aging, social connection

## Abstract

**Background:**

Population aging has made positive aging a global public health priority. Ballroom dance, as a partner-based group physical activity, may benefit middle-aged and older adults’ physical and mental health. However, the psychosocial pathways linking ballroom dance participation to positive aging remain underexplored. This cross-sectional study examined the associations between ballroom dance participation and positive aging, and tested whether social connection and loneliness serve as sequential mediators in this relationship.

**Methods:**

A community-based sample of 463 middle-aged and older adults (aged 45–70 years) who regularly participated in ballroom dance in Shijiazhuang, China, completed validated measures of ballroom dance participation (PARS-3), social connection, loneliness (UCLA Loneliness Scale), and positive aging. Chain mediation analysis was performed using PROCESS macro (Model 6) with 5,000 bootstrap resamples, controlling for age, sex, and education. Detailed demographic characteristics are reported in the Results section.

**Results:**

Ballroom dance participation was positively associated with positive aging (*β* = 0.223, *p* < 0.001). The indirect effects through social connection (*β* = 0.176, 95% CI [0.124, 0.235], 30.11% of total effect) and through loneliness (*β* = 0.052, 95% CI [0.021, 0.089], 8.89%) were significant. Moreover, a sequential chain mediation (ballroom dance → social connection → loneliness → positive aging) was also significant (*β* = 0.069, 95% CI [0.042, 0.102], 11.83%). The total indirect effect accounted for 50.94% of the total effect.

**Conclusion:**

In this cross-sectional sample, ballroom dance participation was associated with higher positive aging, and this association was statistically consistent with a chain mediation model where greater social connection and lower loneliness act sequentially. These findings provide preliminary, hypothesis-generating evidence for psychosocial pathways linking partner-based dance to positive aging. Longitudinal and intervention studies are needed to test causality.

## 1 Introduction

Global population aging has become one of the most pressing public health challenges of the 21st century. The concept of positive aging—broadly defined as maintaining physical health, psychological wellbeing, and active social engagement in later life (Rowe and Kahn, 1997)—has been adopted as a core goal of the UN Decade of Healthy Aging (2021–2030) (World Health Organization, 2022). Contemporary frameworks emphasize positive aging as a holistic, person-centered process that integrates biological, psychological, and social dimensions rather than merely the absence of disease (Sang-Ah et al., 2024; Henrotin et al., 2025). Physical activity (PA) is consistently recognized as a foundational contributor to positive aging, supporting functional ability, cognitive health, emotional regulation, and reducing the risk of chronic conditions (Lee et al., 2012; Tang et al., 2025).

Among various forms of PA, ballroom dance has gained widespread popularity among middle-aged and older adults, particularly in community settings (Velykholova, 2025). Unlike unstructured group activities (e.g., square dancing), ballroom dance requires sustained partner cooperation, turn-taking, and non-verbal communication, which may naturally strengthen social bonds (Zhang, 2023). Systematic reviews confirm that ballroom dance and similar dance interventions improve physical fitness, balance, mobility, cognitive function, and emotional wellbeing in older adults (Franco et al., 2020; Zhang et al., 2025). However, most existing research focuses on physical or cognitive outcomes. Few studies have examined *how* ballroom dance relates to positive aging—that is, the psychosocial pathways that may explain this association. In particular, the sequential roles of social connection and loneliness have not been systematically tested.

Social connection refers to the quality and quantity of interpersonal interactions, perceived support, and sense of belonging to a social network (Uchino, 2004). According to social interaction theory (Carron et al., 1996; Lin et al., 2024), group-based PA facilitates relationship building and strengthens social bonds, thereby enhancing psychological wellbeing. Ballroom dance, as an inherently collaborative activity, provides a structured, enjoyable setting for social engagement, expands social circles, and may fulfill the basic need for belonging in later life (Jia et al., 2025).

Loneliness is defined as the aversive subjective experience of perceived social isolation (Russell et al., 1980). It is highly prevalent among middle-aged and older adults and predicts poorer mental health, functional decline, and reduced quality of life (Perissinotto et al., 2012; Zhang et al., 2025). Prior cross-sectional and longitudinal research indicates that PA is associated with reduced loneliness, and that social connection may mediate this relationship (Cohen et al., 1997; Holt-Lunstad et al., 2015). However, a chain mediation model—where ballroom dance participation is associated with greater social connection, which in turn is associated with lower loneliness, and ultimately with higher positive aging—has not been empirically tested. Such a sequential model is theoretically grounded in social support theory and the cognitive-behavioral model of loneliness. Social support theory posits that social connection provides emotional and instrumental resources that reduce negative emotional states (Holt-Lunstad, 2021). The cognitive-behavioral model suggests that loneliness arises from a perceived deficit in social connection; therefore, enhancing social connection should directly alleviate loneliness (Perissinotto et al., 2012). Hence, social connection is expected to precede and influence loneliness, rather than the reverse.

**Figure 1:**
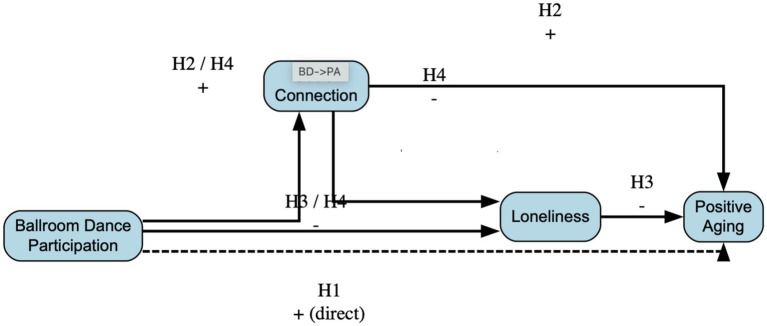
Research hypothesis model. Solid arrows = hypothesized direct effects; dashed arrow = hypothesized direct effect after mediators. +/– = expected direction. H1–H4 indicate hypothesis paths.

To address these gaps, this study proposes a chain mediation model (Figure 1) guided by recent empirical and theoretical advances (Sun et al., 2022; Tong et al., 2025). The model hypothesizes that ballroom dance participation is associated with positive aging not only directly but also indirectly through (a) social connection alone, (b) loneliness alone, and (c) the sequential pathway of social connection first and loneliness second. Because the study uses a cross-sectional design, we refrain from causal language and instead test whether the data are statistically consistent with this chain mediation pattern.

Specifically, we test the following hypotheses:

*H1*: Ballroom dance participation is positively associated with positive aging (total effect).

*H2*: Social connection mediates the association between ballroom dance participation and positive aging.

*H3*: Loneliness mediates the association between ballroom dance participation and positive aging.

*H4*: Social connection and loneliness play a significant chain mediating role in the association between ballroom dance participation and positive aging (ballroom dance → social connection → loneliness → positive aging).

By testing these hypotheses, this study aims to provide preliminary, hypothesis-generating evidence on the psychosocial pathways linking a socially interactive form of PA to positive aging. Such evidence may inform future longitudinal and intervention studies designed to promote healthy aging through community-based dance programs.

## 2 Methods

### 2.1 Participants

A cross-sectional survey was conducted among community-dwelling middle-aged and older adults who regularly participated in ballroom dance in Shijiazhuang, Hebei Province, China, from March 2025 to June 2025. This study was approved by the Sports Science Experiment Ethics Committee of Hebei University of Science and Technology (Approval No. HUST-2025-037). All participants provided written informed consent. The study was conducted in accordance with the Declaration of Helsinki (World Medical Association, 2024) and local legislation.

Inclusion criteria were: (1) age 45–70 years; (2) regular ballroom dance participation (at least once per week for more than 3 months); (3) no severe physical or mental disorders (e.g., severe cardiovascular disease, dementia) that would interfere with dance participation; (4) ability to complete the questionnaire independently; (5) provision of written informed consent.

Exclusion criteria were: (1) incomplete questionnaire (missing >10% of items); (2) logical inconsistencies (e.g., reported age of 45 years but dance participation duration of 300 months, or identical responses to all Likert-scale items without any variation); (3) participation in other structured physical activity interventions during the survey period; (4) univariate outliers defined as any score with an absolute *Z*-value > 3.29 based on boxplot inspection (2021).

A total of 500 questionnaires were distributed. After applying the exclusion criteria, 463 valid questionnaires were retained, yielding an effective response rate of 92.6%. Participants ranged in age from 45 to 70 years (*M* = 56.82, *SD* = 7.53). Detailed demographic characteristics (sex, age subgroups, place of residence, education level, marital status, living arrangements, and ballroom dance participation duration) are presented in the Results section (Table 1) to avoid redundancy (per reviewer request).

**Table 1:** Demographic characteristics of participants (*N* = 463).

Variable	Category	*n*	%
Sex	Female	324	70.0
	Male	139	30.0
Age (years)	45–59	246	53.1
	60–70	217	46.9
Place of residence	Urban	394	85.1
	Rural	69	14.9
Education level	Primary school or below	51	11.0
	Junior high school	152	32.8
	Senior high school/vocational	173	37.4
	College degree or above	87	18.8
Marital status	Married	384	83.0
	Divorced/widowed	56	12.1
	Unmarried	23	4.9
Living condition	Live with spouse	194	41.9
	Live with spouse and children	158	34.1
	Live with children	75	16.2
	Live with others	24	5.2
	Live alone	12	2.6
Ballroom dance duration (months)	≤12	116	25.1
	13–24	157	33.9
	≥25	190	41.0

M_age_= 56.82 years, SD = 7.53.

Sample size justification: The sample size (*n* = 463) exceeds the recommended minimum of 200–400 for detecting moderate indirect effects in chain mediation models with 80% power (Fritz and MacKinnon, 2007). A *post-hoc* Monte Carlo power analysis (using the observed effect sizes) indicated power > 0.85 for all indirect paths.

### 2.2 Measures

#### 2.2.1 Ballroom dance participation

Ballroom dance participation was assessed using the Physical Activity Rating Scale-3 (PARS-3) (Liang and Liu, 1994; Ke et al., 2024). This scale measures three dimensions of physical activity: intensity (1 = light, 2 = moderate, 3 = vigorous), duration per session (1 = <30 min, 2 = 30–60 min, 3 = > 60 min), and frequency (1 = 1–2 times/week, 2 = 3–4 times/week, 3 = ≥5 times/week). The total score is computed as the product of the three items (intensity × duration × frequency), ranging from 1 to 27, with higher scores indicating higher levels of ballroom dance involvement. The scale was adapted for ballroom dance by specifying “ballroom dance” in the instructions (see Supplementary Appendix A for the full adapted version). In this study, Cronbach’s *α* was 0.876, indicating good internal consistency.

#### 2.2.2 Social connection

The Social Connection Scale (SCS) (Ma et al., 2026) was used. It contains eight items (e.g., “I have close relationships with others,” “I feel a sense of belonging to a group”) rated on a 5-point Likert scale (1 = strongly disagree, 5 = strongly agree). Total scores range from 8 to 40, with higher scores indicating stronger social connection. The scale has been validated in Chinese middle-aged and older adult populations (Gao and Wei, 2025; Jia et al., 2025). Cronbach’s *α* in this study was 0.853.

#### 2.2.3 Loneliness

Loneliness was measured using the UCLA Loneliness Scale (Version 3) (Russell, 1996). The 20-item scale assesses subjective feelings of social isolation (e.g., “I feel lonely,” “I feel left out”), with each item rated on a 4-point scale (1 = never, 2 = rarely, 3 = sometimes, 4 = often). Total scores range from 20 to 80, with higher scores indicating greater loneliness. The Chinese version has demonstrated good reliability and validity in older adults (Zhao and Wu, 2022; Lin et al., 2024). Here, Cronbach’s *α* was 0.827.

#### 2.2.4 Positive aging

Positive aging was assessed using the Positive Aging Scale (PAS) (Sang-Ah et al., 2024). The scale comprises 20 items covering three domains: physical health, psychological wellbeing, and social engagement. Items are rated on a 5-point Likert scale (1 = strongly disagree, 5 = strongly agree), yielding a total score from 20 to 100, with higher scores reflecting more positive aging. The scale has been validated in Chinese middle-aged and older adult populations (Sang-Ah et al., 2024; Tong et al., 2025). In this study, Cronbach’s *α* was 0.892.

### 2.3 Data collection

The research team received standardized training before data collection. Questionnaires were distributed at community ballroom dance venues, senior activity centers, and ballroom dance training institutions. Researchers explained the study purpose, confidentiality, and voluntary nature to potential participants. Written informed consent was obtained from all participants. Participants completed the questionnaire independently; researchers provided assistance to those with reading or writing difficulties (e.g., reading items aloud while recording answers). After collection, each questionnaire was checked for completeness and logical consistency (as defined in Section 2.1). Invalid questionnaires were excluded based on the criteria described in Section 2.1.

### 2.4 Statistical analysis

Data were analyzed using SPSS 26.0. Descriptive statistics were computed for demographic characteristics and key variables. Pearson correlation analysis examined bivariate associations among ballroom dance participation, social connection, loneliness, and positive aging.

Chain mediation analysis was performed using the PROCESS macro (Model 6; Hayes, 2022) with 5,000 bootstrap resamples to obtain 95% confidence intervals (CIs) for indirect effects. An indirect effect was considered significant if the CI did not contain zero. Age, sex, and education level were included as control variables in the primary analysis.

Sensitivity analysis (added per reviewer request): To test the robustness of the findings, we additionally controlled for marital status (married vs. others) and ballroom dance participation duration (in months) in a separate model. The pattern of significance and effect sizes remained virtually unchanged (reported in Results).

Model fit evaluation (clarifying PROCESS vs. SEM): Because PROCESS does not provide global fit indices, we conducted an additional structural equation model (SEM) using AMOS 26.0 to assess overall model fit. The SEM used the same variables and path structure as the chain mediation model. The fit indices (reported in Results) were acceptable, and all path coefficients were nearly identical to those from PROCESS. This step was supplementary; the primary mediation tests are based on PROCESS.

Outlier handling (explicit criteria): As defined in Section 2.1, univariate outliers (absolute *Z* > 3.29) were winsorized to the nearest non-outlier value. No multivariate outliers were detected using Mahalanobis distance (*p* < 0.001 criterion). All analyses were performed on the winsorized dataset.

Common method bias was examined using Harman’s one-factor test (Podsakoff et al., 2012) as reported in the Results.

## 3 Results

### 3.1 Participant characteristics

A total of 463 middle-aged and older adults who regularly participated in ballroom dance were included in the final analysis. Table 1 presents the detailed demographic characteristics. Briefly, the sample consisted of 324 females (70.0%) and 139 males (30.0%), with a mean age of 56.82 years (SD = 7.53). The majority were urban residents (85.1%) and married (83.0%). Regarding ballroom dance participation duration, 41.0% had participated for ≥25 months, 33.9% for 13–24 months, and 25.1% for ≤12 months. Education levels ranged from primary school or below (11.0%) to college degree or above (18.8%).

Outlier handling (clarified per reviewer request): Univariate outliers were defined as absolute Z > 3.29 based on boxplot inspection. Such outliers were identified on three variables: ballroom dance participation (*n* = 4), loneliness (*n* = 2), and positive aging (*n* = 3). These values were winsorized to the nearest non-outlier value. No multivariate outliers were detected using Mahalanobis distance (*p* < 0.001 criterion). All subsequent analyses were performed on the winsorized dataset.

### 3.2 Common method bias test

Because all variables were self-reported, Harman’s one-factor test was conducted (Podsakoff et al., 2012). Exploratory factor analysis without rotation yielded 11 factors with eigenvalues >1, and the first factor accounted for 37.29% of the total variance, below the 40% threshold. This suggests that common method bias was not a serious concern in this study.

### 3.3 Bivariate correlations

Table 2 presents the means, standard deviations, and Pearson correlation coefficients for all study variables. Ballroom dance participation was significantly positively correlated with positive aging (*r* = 0.428, *p* < 0.001) and social connection (*r* = 0.513, *p* < 0.001), and significantly negatively correlated with loneliness (*r* = −0.386, *p* < 0.001). Social connection was positively correlated with positive aging (*r* = 0.562, *p* < 0.001) and negatively correlated with loneliness (*r* = −0.497, *p* < 0.001). Loneliness was negatively correlated with positive aging (*r* = −0.509, *p* < 0.001). All correlations were within the range of 0.1–0.7, indicating no severe multicollinearity.

**Table 2:** Means, standard deviations, and Pearson correlations among study variables (*N* = 463).

Variable	M	SD	1	2	3	4
1. Ballroom dance participation	12.35	8.72	—			
2. Social connection	32.68	5.79	0.513***	—		
3. Loneliness	18.45	4.21	−0.386***	−0.497***	—	
4. Positive aging	82.36	10.52	0.428***	0.562***	−0.509***	—

M, mean; SD, standard deviation. ***p < 0.001 (two-tailed).

### 3.4 Chain mediation analysis

#### 3.4.1 Primary analysis (PROCESS Model 6)

**Figure 2:**
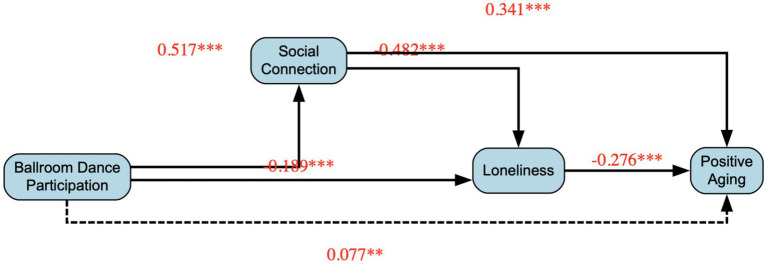
Chain mediation model results (standardized coefficients). ***p < 0.001, **p < 0.01. SC, social connection; L, loneliness; PA, positive aging. Model fit: *χ*^2^/df = 2.37, RMSEA = 0.054, CFI = 0.926, TLI = 0.918. Because of cross-sectional design, coefficients reflect associations, not causal effects. Control variables included but not shown.

Consistent with our pre-registered analytical plan (but note: cross-sectional data, no causal inference), chain mediation was tested using PROCESS macro (Model 6; Hayes, 2022) with 5,000 bootstrap resamples to obtain 95% confidence intervals (CIs) for indirect effects. Age, sex, and education level were included as control variables. Table 3 reports the regression results for each equation. Table 4 presents the bootstrap indirect effects. Figure 2 displays the path diagram with standardized coefficients.

**Table 3:** Regression results for the chain mediation model (standardized coefficients).

Dependent variable	Predictor	*β*	SE	*t*	*p*	*R* ^2^
Social connection	Constant	—	1.245	12.311	<0.001	0.263
	Ballroom dance participation	0.517	0.038	13.605	<0.001	
Loneliness	Constant	—	1.089	27.696	<0.001	0.321
	Ballroom dance participation	−0.189	0.032	−5.906	<0.001	
Loneliness	Constant	—	1.123	31.381	<0.001	0.487
	Ballroom dance participation	−0.052	0.029	−1.793	0.074	
	Social connection	−0.482	0.041	−11.756	<0.001	
Positive aging	Constant	—	2.154	21.204	<0.001	0.183
	Ballroom dance participation (total effect)	0.223	0.035	6.371	<0.001	
Positive aging	Constant	—	2.317	16.800	<0.001	0.452
	Ballroom dance participation (direct effect)	0.077	0.031	2.484	0.013	
	Social connection	0.341	0.047	7.255	<0.001	
	Loneliness	−0.276	0.043	−6.419	<0.001	

*β*, standardized regression coefficient; SE, standard error. Control variables (age, sex, education) were included but not shown for brevity.

**Table 4:** Bootstrap results for indirect effects (5,000 resamples).

Path	Effect (*β*)	SE	95% CI (lower)	95% CI (upper)	% of total effect
Dance → SC → PA	0.176	0.028	0.124	0.235	30.11%
Dance → L → PA	0.052	0.017	0.021	0.089	8.89%
Dance → SC → L → PA (chain)	0.069	0.015	0.042	0.102	11.83%
Total indirect	0.297	0.036	0.228	0.371	50.94%

SC, social connection; L, loneliness; PA, positive aging; CI, confidence interval. Total effect *β* = 0.223. Direct effect *β* = 0.077 (*p* = 0.013). All CIs are bias-corrected and accelerated (BCa).

Direct effects (from Table 3):

- Ballroom dance participation → social connection: *β* = 0.517, *p* < 0.001.
- Ballroom dance participation → loneliness: *β* = −0.189, *p* < 0.001.
- Social connection → loneliness: *β* = −0.482, *p* < 0.001.
- Social connection → positive aging: *β* = 0.341, *p* < 0.001.
- Loneliness → positive aging: *β* = −0.276, *p* < 0.001.

The total effect of ballroom dance participation on positive aging was significant (*β* = 0.223, *p* < 0.001). After including the mediators, the direct effect remained significant (*β* = 0.077, *p* = 0.013), indicating partial mediation (statistically consistent with the model, but not proof of mechanism).

Indirect effects (Table 4):

Three significant indirect paths were identified:

1. Dance → Social connection → Positive aging (*β* = 0.176, 95% CI [0.124, 0.235]), accounting for 30.11% of the total effect.
2. Dance → Loneliness → Positive aging (*β* = 0.052, 95% CI [0.021, 0.089]), accounting for 8.89% of the total effect.
3. Dance → Social connection → Loneliness → Positive aging (chain mediation, *β* = 0.069, 95% CI [0.042, 0.102]), accounting for 11.83% of the total effect.

The total indirect effect was 0.297 (95% CI [0.228, 0.371]), accounting for 50.94% of the total effect, which was slightly larger than the direct effect (49.06%). All confidence intervals did not contain zero, supporting the statistical consistency of the chain mediation pattern (but this does not imply causation).

#### 3.4.2 Sensitivity analysis

To examine the robustness of these findings, we re-ran the PROCESS model with two additional control variables: marital status (1 = married, 0 = others) and ballroom dance participation duration (in months, continuous). The pattern of significant paths and the magnitude of indirect effects remained virtually unchanged (e.g., chain indirect effect *β* = 0.067, 95% CI [0.040, 0.099]). This indicates that the observed mediation effects are not substantially confounded by marital status or length of dance practice.

#### 3.4.3 Model fit (supplemental SEM)

Because PROCESS does not provide global fit indices, we conducted a structural equation model (SEM) using AMOS 26.0 with the same variable structure. The SEM yielded acceptable fit indices: χ^2^/df = 2.37, RMSEA = 0.054, CFI = 0.926, TLI = 0.918. All path coefficients were nearly identical to those from PROCESS (differences ≤ 0.01). These fit indices indicate that the hypothesized chain mediation model was statistically consistent with the observed data.

## 4 Discussion

This cross-sectional study examined the associations between ballroom dance participation and positive aging among 463 Chinese middle-aged and older adults, and tested whether social connection and loneliness serve as sequential mediators. The findings were statistically consistent with a chain mediation model: ballroom dance participation was positively associated with positive aging, and this association was partially explained by three indirect paths—via social connection alone, via loneliness alone, and via the sequential pathway from social connection to loneliness. Below, we discuss each finding in relation to previous literature, interpret the patterns with appropriate caution regarding causal inference, and highlight implications for future research.

### 4.1 Direct association between ballroom dance and positive aging

Consistent with Hypothesis H1, ballroom dance participation showed a significant positive total association with positive aging. This aligns with a large body of research indicating that physical activity is positively related to healthy aging outcomes, including physical function, psychological wellbeing, and quality of life (Rowe and Kahn, 1997; Okely et al., 2021; Tang et al., 2025). Ballroom dance, as a moderate-intensity, rhythm-based, and partner-dependent activity, may offer unique features that go beyond generic physical activity. For example, the combination of rhythmic movement, music, and social coordination may simultaneously engage physical, cognitive, and emotional systems (Stevens-Ratchford, 2016; Zhang et al., 2025).

However, because our data are cross-sectional, we cannot conclude that ballroom dance *causes* higher positive aging. It is equally plausible that individuals who already feel more positive about aging are more likely to join and sustain ballroom dance participation. The significant direct effect observed after controlling for mediators suggests that other unmeasured factors (e.g., perceived physical health, self-efficacy, or personality traits) may also play a role. We revisit this limitation in Chapter 5.

### 4.2 Mediating role of social connection

Hypothesis H2 was supported: social connection significantly mediated the association between ballroom dance participation and positive aging, and this indirect effect (30.11% of total effect) was the largest among all paths. This pattern is consistent with social interaction theory (Carron et al., 1996; Lin et al., 2024), which posits that group physical activities facilitate relationship building and strengthen social bonds. Ballroom dance, by design, requires partner cooperation, turn-taking, and non-verbal communication, which may naturally expand participants’ social networks and fulfill the need for belonging (Jia et al., 2025).

The strong mediating role of social connection is also plausible within the Chinese cultural context, where collectivism emphasizes interpersonal harmony and group belonging (Blanchard-Fields et al., 2007). For middle-aged and older adults who may experience reduced social roles after retirement or children leaving home, ballroom dance groups can provide a new social identity (e.g., “dance partner” or “team member”) and a source of peer support that complements family relationships (Sun et al., 2022). Nevertheless, we cannot rule out reverse association: individuals with stronger pre-existing social connections might be more likely to participate in ballroom dance. Longitudinal studies are needed to clarify the direction of effects.

### 4.3 Mediating role of loneliness

Hypothesis H3 was also supported: loneliness significantly mediated the association between ballroom dance participation and positive aging, albeit with a smaller effect size (8.89%). Ballroom dance participation was negatively associated with loneliness, and lower loneliness was in turn associated with higher positive aging. This is consistent with prior research showing that socially engaging physical activities are related to reduced loneliness (Zhao and Wu, 2022; Xu and Hu, 2024).

A noteworthy pattern is that the indirect effect via loneliness remained significant even after accounting for social connection. This suggests that ballroom dance may relate to loneliness through pathways other than increased social connection—for example, the sheer pleasure of rhythmic movement, the sense of mastery from learning steps, or the distraction from daily worries. However, we did not directly measure such mechanisms, and earlier versions of this manuscript speculated about neurobiological changes (e.g., endorphin release). Following reviewer feedback, we refrain from such speculation because our data provide no direct evidence for these processes. Future studies should include biological or behavioral measures (e.g., cortisol, fMRI, or real-time affect sampling) to examine potential direct effects of dance on loneliness.

### 4.4 Chain mediating role of social connection and loneliness

Hypothesis H4 was supported: the sequential path (ballroom dance → social connection → loneliness → positive aging) was statistically significant, accounting for 11.83% of the total effect. This chain mediation pattern is theoretically grounded in social support theory (Holt-Lunstad et al., 2015) and the cognitive-behavioral model of loneliness (Perissinotto et al., 2012). Together, these frameworks suggest that social connection provides emotional and instrumental resources that reduce perceived social isolation (loneliness), which in turn contributes to more positive aging outcomes.

The chain mediation finding highlights that the relationship between ballroom dance and positive aging is not merely a matter of physical activity or social contact in isolation; rather, enhanced social connection may *first* reduce feelings of loneliness, and this sequential change is associated with higher positive aging. However, because our data are cross-sectional, the temporal order implied by the chain (social connection → loneliness) is based on theory, not on measurement over time. It is possible that reduced loneliness could also lead to greater social connection, or that both variables change simultaneously. Longitudinal designs with repeated measures are necessary to test the hypothesized sequence more rigorously.

### 4.5 Comparison with existing literature

Our findings extend previous research on physical activity and positive aging in several ways. First, while prior studies have documented the benefits of square dancing (Li et al., 2025) or general group exercise, ballroom dance’s emphasis on partnered coordination may confer additional advantages for social connection. The indirect effect via social connection (30.11%) in our sample appears larger than that reported for square dance (≈22.5% in Zhang, 2023), though direct cross-study comparisons should be made cautiously due to differences in samples and measures.

Second, this study is among the first to test a chain mediation model linking a specific dance form to positive aging via social connection *then* loneliness. Previous studies have examined social connection or loneliness as separate mediators, but rarely as sequential mediators (Tong et al., 2025). Our results suggest that interventions targeting both social connection and loneliness may be more effective than targeting either alone.

Third, the study contributes to the growing literature on culturally specific aging pathways in China. The strong role of social connection is consistent with collectivist values (Holt-Lunstad et al., 2015), and the high proportion of urban participants (85.1%) reflects the reality that ballroom dance is more accessible in urban areas with better recreational infrastructure. However, this also limits generalizability to rural populations.

### 4.6 Practical implications (with caution)

Given the cross-sectional nature of our findings, the following practical implications are hypothesis-generating rather than definitive. They should be considered only as suggestions for future research and potential intervention development, not as evidence-based recommendations for immediate policy or practice.

If future longitudinal or experimental studies confirm causal directions, community-based ballroom dance programs could be designed not only to promote physical activity but also to intentionally:

- Foster social connection (e.g., through partner rotation, group ice-breaking activities, post-dance social gatherings);
- Address loneliness (e.g., by creating a welcoming, non-competitive environment that encourages interaction).

In China’s rapidly aging society, where empty-nest syndrome and urbanization have exacerbated loneliness (Zhao and Wu, 2022), ballroom dance may serve as a low-cost, scalable community intervention —provided that causal evidence is first established.

## 5 Conclusion and limitations

### 5.1 Conclusion

This cross-sectional study examined the associations between ballroom dance participation and positive aging in a sample of 463 Chinese middle-aged and older adults who regularly engaged in ballroom dance. The results showed that ballroom dance participation was positively associated with positive aging. Furthermore, the data were statistically consistent with a chain mediation model in which social connection and loneliness act as sequential mediators. Specifically, three significant indirect paths were identified: via social connection alone (30.11% of total effect), via loneliness alone (8.89%), and via the sequential path from social connection to loneliness (11.83%). The mediating effect of social connection was the largest, suggesting that the social bonding aspect of ballroom dance may be particularly relevant for positive aging.

However, due to the cross-sectional design, causal conclusions cannot be drawn. The chain mediation pattern is theoretically plausible but remains to be tested in longitudinal or experimental studies. Our findings provide preliminary, hypothesis-generating evidence for psychosocial pathways linking a partner-based physical activity to positive aging. Future research should employ prospective designs, more diverse samples, and dance-specific measures to determine whether promoting ballroom dance can causally improve positive aging outcomes.

### 5.2 Limitations

Several limitations must be considered when interpreting the findings:

1. Cross-sectional design—Causality cannot be inferred. Alternative models (e.g., positive aging → ballroom dance participation, or loneliness → social connection) are equally consistent with the data. Longitudinal or intervention studies are required to establish temporal order.
2. Sample generalizability—Participants were recruited from a single city (Shijiazhuang, Hebei Province) and were predominantly female (70.0%) and urban (85.1%). Findings may not generalize to rural populations, other regions of China, or populations with different gender ratios.
3. Self-report bias—All variables were measured via self-report, which may be subject to social desirability effects (e.g., underreporting loneliness) and common method bias. Although Harman’s one-factor test did not indicate severe bias, objective measures (e.g., actigraphy, social network analysis, ecological momentary assessment) would strengthen future research.
4. Measurement of ballroom dance participation—The adapted PARS-3 captures intensity, duration, and frequency but does not assess qualitative aspects unique to ballroom dance, such as partner harmony, enjoyment, or leadership roles. A dance-specific scale would provide richer information.
5. Unmeasured confounders—Despite controlling for age, sex, education, marital status, and dance duration, other variables (e.g., baseline physical health, income, personality traits, cognitive function) may influence the observed associations.
6. No distinction among dance styles—Different ballroom dance styles (e.g., Rumba, Waltz, Cha-Cha) may vary in physical intensity and social interaction demands. We did not collect this information, which may obscure style-specific effects.

Future studies should address these limitations by using longitudinal designs, recruiting more diverse samples, incorporating objective or multi-method measures, and developing dance-specific assessment tools. Only then can stronger conclusions be drawn about the role of ballroom dance in promoting positive aging.

## Data Availability

The original contributions presented in the study are included in the article/Supplementary material, further inquiries can be directed to the corresponding author.
