# Supplementary material for: Ballroom dance and positive aging among middle-aged and older adults: a chain mediation model of social connection and loneliness
Source: Front Psychol. 2026 May 29;17:1855200. doi: 10.3389/fpsyg.2026.1855200 (PMC13259695; doi:10.3389/fpsyg.2026.1855200)
Supplement: Supplementary file 1 [file Data_Sheet_1.PDF]

Appendix A: Adapted PARS-3 for Ballroom Dance Participation

Instructions: Please answer the following three questions based **only on your ballroom dance activities** over the past month. For each question, select the option that best describes your typical participation.

Item 1: Intensity of ballroom dance

Choose the level that best describes the usual intensity of your ballroom dance practice.

Score	Description
1	**Light** – No sweating, no noticeable increase in heart rate or breathing
2	**Moderate** – Mild sweating, slight increase in heart rate and breathing (e.g., able to talk but not sing)
3	**Vigorous** – Obvious sweating, significant increase in heart rate and breathing (e.g., difficult to talk)

Item 2: Duration per session

How long does each ballroom dance session typically last?

Score	Description
1	Less than 30 minutes
2	30 to 60 minutes
3	More than 60 minutes

Item 3: Frequency

How often do you participate in ballroom dance per week?

Score	Description
1	1–2 times per week
2	3–4 times per week
3	5 or more times per week

Total Score Calculation:

Total score = Intensity score × Duration score × Frequency score

- Range: 1 to 27

- Higher total scores indicate higher levels of ballroom dance participation.

Note to reviewers and readers: This adapted version of the PARS-3 (Liang & Liu, 1994; Ke et al., 2024) specifies “ballroom dance” in the instructions. The original scale has been validated in Chinese populations for general physical activity. The adaptation does not alter item wording or scoring; only the activity context is specified.
